# A central role for aspartate in *Mycobacterium tuberculosis* physiology and virulence

**DOI:** 10.3389/fcimb.2013.00068

**Published:** 2013-10-24

**Authors:** Alexandre Gouzy, Yannick Poquet, Olivier Neyrolles

**Affiliations:** ^1^Centre National de la Recherche Scientifique, Institut de Pharmacologie et de Biologie StructuraleToulouse, France; ^2^Institut de Pharmacologie et de Biologie Structurale, Université de Toulouse, Université Paul SabatierToulouse, France

**Keywords:** *Mycobacterium tuberculosis*, macrophage, phagosome, aspartate, nitrogen

The tuberculosis (TB) bacillus, *Mycobacterium tuberculosis*, is a facultative intracellular pathogen that multiplies inside macrophages, in which it resides within a specialized compartment, the phagosome, where nutrient sources are likely limited. A number of studies provided compelling evidence that *M. tuberculosis* has the ability to exploit host-derived carbon sources, such as triglycerides, cholesterol and glucose, and to proliferate inside host cells (Mckinney et al., 2000; Pandey and Sassetti, 2008; Daniel et al., 2011; Marrero et al., 2013). In addition to carbon, nitrogen is an essential constituent of all living organisms. As compared to carbon requirements, little is known about the nature of nitrogen-containing molecules utilized by the TB bacillus during infection. We recently discovered that nitrogen incorporation from exogenous aspartate is required for host colonization by *M. tuberculosis* (Gouzy et al., 2013). This study highlights, for the first time, the potential of amino acids, and aspartate in particular, as a major nitrogen reservoir supporting *M. tuberculosis* virulence *in vivo*. It also opens a series of questions to be addressed in the future.

Chief among these questions is the origin and molecular mechanism(s) of transport allowing mycobacterial access to aspartate inside host cells. TB lesions are enriched in aspartate, and this likely relies on global metabolic changes in immune cells during granuloma formation (Somashekar et al., 2011). How nutrients, such as aspartate, access the *M. tuberculosis* phagosome during infection is an intriguing issue. Using mass spectrometry imaging, we further showed that aspartate can access the mycobacterial phagosome, at least in vitro (Gouzy et al., 2013). In mammalian cells, aspartate uptake relies on transporters of the solute carriers (SLC) superfamily, and in particular on those of the high-affinity glutamate and neutral amino acid (SLC1) and of the cationic amino acid (SLC7) transporter families. Among these transporters, SLC1A2 was reported to mediate aspartate transport inside macrophages (Rimaniol et al., 2001; Ye et al., 2010). Interestingly, we reported the expression of the *slc1a2* gene is increased in human macrophages upon *M. tuberculosis* infection (Tailleux et al., 2008). Increased expression of this transporter could impact considerably the ability of *M. tuberculosis* to multiply inside macrophages, as it is the case for the neutral amino acid transporter SLC1A5 in the context of *Francisella tularensis* and *Legionella pneumophila* infection (Wieland et al., 2005; Barel et al., 2012). These studies suggested the transport of yet to be identified amino acid(s) is important to sustain intracellular multiplication of these two bacterial species. Whether SLC1A2 and/or other members of the SLC superfamily allow *M. tuberculosis* to access aspartate and multiply inside host cells remains to be evaluated.

Another issue raised by our study is: how is nitrogen transferred from aspartate to other nitrogen-containing molecules in *M. tuberculosis*? Once acquired by the bacillus through its unique aspartate importer AnsP1, we showed this amino acid species mostly serves as a nitrogen provider and barely enters carbon metabolism through the Krebs cycle (Gouzy et al., 2013). Nitrogen assimilation from aspartate must rely on transamination steps allowing the transfer of aspartate-derived nitrogen to glutamate, which in turn, together with glutamine, provides nitrogen to most of biosynthesis pathways. In *M. tuberculosis*, two aspartate transaminases, called AspB and AspC, are predicted to mediate nitrogen transfer from aspartate to glutamate (Cole et al., 1998). Interestingly, the *aspC* gene is thought to be essential in *M. tuberculosis* (Sassetti et al., 2003), which may indicate that AspC is involved mostly in aspartate biosynthesis, rather than in aspartate catabolism and glutamate synthesis. Genetic inactivation of *aspC* may thus result in aspartate auxotrophy. The *aspB* gene, on the opposite, is not essential *in vitro* (Sassetti et al., 2003). Interestingly, *aspB* is expressed at higher level in bacteria withstanding conditions mimicking the intracellular environment (Fisher et al., 2002). AspB may thus be involved in aspartate-derived nitrogen assimilation during infection, and may be required for *M. tuberculosis* virulence. Noteworthy, AspB and AspC possess two homologue proteins, called Rv0858c and Rv1178, that are predicted to act as aspartate aminotransferases, and that might provide alternative pathways for aspartate-derived nitrogen assimilation in the TB bacillus (Cole et al., 1998).

Finally, our study suggests aspartate is an important metabolite required for mycobacterial virulence, and this raises several questions regarding the therapeutic potential of inhibitors of enzymes using aspartate as a substrate. In addition to serve as a nitrogen provider, aspartate possesses non-redundant and essential functions in bacterial metabolism. As a consequence, several enzymes involved in aspartate metabolism, namely the aspartate decarboxylase PanD, the aspartokinase ASK, and the aspartate-β-semialdehyde dehydrogenase ASD, are considered promising targets for novel antituberculous compounds (Shafiani et al., 2005; Gopalan et al., 2006; Chaitanya et al., 2010; Sharma et al., 2012a,b). PanD is the sole enzyme in *M. tuberculosis* mediating the decarboxylation of aspartate, which allows the formation of vitamin B5, a precursor of the coenzyme A (CoA) co-factor. CoA is an acyl-carrier co-enzyme involved in fatty acid metabolism and is essential for the viability of several microorganisms, including *M. tuberculosis* (Ambady et al., 2012). Consequently, deletion of the *panD* gene was shown to result in a severe attenuation of *M. tuberculosis* virulence in the mouse model (Sambandamurthy et al., 2002). Another essential pathway relying on aspartate metabolism is the synthesis of amino acids belonging to the aspartate amino acid family, namely methionine, threonine and isoleucine, and involving the ASK and ASD enzymes, that play a unique and essential part in aspartate incorporation into these biosynthesis pathways. Furthermore, in addition to their role in the biosynthesis of these amino acids, both enzymes are involved in the synthesis of diaminopimelic acid, an intermediate of the lysine biosynthesis pathway, which is an essential constituent of the bacterial cell wall constituent peptidoglycan. As a consequence, ASK and ASD are attractive drug targets for new antituberculous molecules since i/ they possess no homologue in humans, and ii/ their inhibition in *M. tuberculosis* may cause both amino acid auxotrophy and cell wall destruction (Shafiani et al., 2005; Chaitanya et al., 2010).

In conclusion, we believe aspartate acquisition and assimilation pathways should be further considered as promising targets for novel anti-TB therapies.
